# LINE-1, the NORth star of nucleolar organization

**DOI:** 10.1101/gad.352583.124

**Published:** 2025-02-01

**Authors:** Misaki Matsuo, Gael Cristofari

**Affiliations:** Institute for Research on Cancer and Aging of Nice (IRCAN), Institut National de la Santé et de la Recherche Médicale (INSERM), Centre National de la Recherche Scientifique (CNRS), University Cote d'Azur, Nice 06107, France

**Keywords:** human embryonic stem cells, LINE1, nucleolus, rDNA arrays

## Abstract

Long interspersed element-1 (LINE-1) retrotransposons are abundant transposable elements in mammals and significantly influence chromosome structure, chromatin organization, and 3D genome architecture. In this issue of *Genes & Development*, Ataei et al. (doi:10.1101/gad.351979.124) identify a homininae-specific LINE-1 element within nucleolar organizer regions (NORs) that is specifically transcribed in naïve human embryonic stem cells. Deletion or silencing of this element disrupts nucleolar organization and function and alters cellular identity. These findings provide novel insights into the role of retrotransposons in genome organization and suggest that individual LINE-1 elements may have evolved specialized roles.

## Abstract (precis)

In this Outlook, Matsuo and Cristofari discuss a study in this issue of *Genes & Development* by Ataei et al. that shows that LINE1 retrotransposons within nucleolar distal junction regions control nucleolar organization and function in hESCs. Matsuo and Cristofari emphasize the functional influence of LINE1 retrotransposons in regulating genome architecture and transcriptional dynamics.

Long interspersed element-1 (LINE-1) retrotransposons are abundant transposable elements in mammalian genomes, representing almost one-fifth of the human or murine genome. Their high copy number results from retrotransposition, a copy and paste mechanism by which LINE-1 elements have spread throughout the chromosomes, introducing *cis*-regulatory elements at new genomic locations. Although only a relatively small fraction of LINE-1 elements remains transpositionally active in modern humans, leading to genetic variation and occasionally genetic diseases, their past activity has profoundly shaped higher-order genome architecture and function. LINE-1 elements accumulate in B compartments (chromatin domains associated with repressed chromatin), contributing to their establishment and/or reinforcement and clustering in the nuclear and nucleolar peripheries (Solovei et al. 2016; Lu et al. 2021). Consistently, LINE-1 elements are strongly associated with Giemsa/quinacrine-positive bands (G/Q bands), which represent heterochromatin, on metaphase chromosomes (Solovei et al. 2016). At a more regional scale, LINE-1 elements frequently contain CTCF binding motifs, which play a crucial role in establishing 3D genome architecture by acting as loop anchors and contributing to topologically associating domain (TAD) formation. Consequently, CTCF motifs within LINE-1 elements can facilitate the dynamic regulation of gene expression in a cell type- or lineage-specific manner, drive regulatory innovation, and enable the emergence of novel phenotypes (Choudhary et al. 2023). LINE-1 elements can be transcribed from an internal promoter within their 5′ UTR. Although LINE-1 RNA encodes the machinery required for retrotransposition and is an essential intermediate in this process, it also functions as a nuclear long noncoding RNA (lncRNA) with diverse roles in genome organization. Indeed, in situ hybridization with Cot-1 DNA repeat probes (which detect abundant nuclear LINE-1-derived RNA species) has shown an association between repetitive RNA and the maintenance of interphase chromosome territories and euchromatin (Hall et al. 2014). Interestingly, these LINE-1 transcripts mostly act in *cis*, colocalizing with their chromosome of origin and associating with the nuclear matrix. Similarly, LINE-1 RNA regulates timely chromatin accessibility in mouse preimplantation embryo, thereby promoting early development (Jachowicz et al. 2017; Percharde et al. 2018). In this issue of *Genes & Development*, Ataei et al. (2025) now demonstrate how a LINE-1 element (adjacent to ribosomal DNA repeats) and its transcript influence nucleolar organization and function.

The nucleolus is a large nuclear compartment responsible for rRNA synthesis and ribosome biogenesis. Recent studies have shown that nucleoli also control early development gene expression, particularly the exit from the 2 cell (2C) state in mouse embryos or the equivalent 8 cell (8C) stage in human embryos (Yu et al. 2021; Xie et al. 2022). Inversely, nucleolar stress promotes a 2C-like state. Nucleolus formation and function are regulated by nucleolar organizer regions (NORs) on the p-arms of human acrocentric chromosomes (chromosomes 13, 14, 15, 21, and 22) (van Sluis et al. 2019). NORs contain tandem arrays of ribosomal RNA genes flanked by proximal junctions (PJs) and distal junctions (DJs) on the centromeric and telomeric sides of the chromosome arm, respectively (Fig. 1). Notably, DJs contain large inverted repeats (>100 kb) that drive the synthesis of a set of lncRNAs called disnor RNAs, which are essential for nucleolar function, through molecular mechanisms that remain to be fully elucidated (van Sluis et al. 2019).

**Figure 1.**
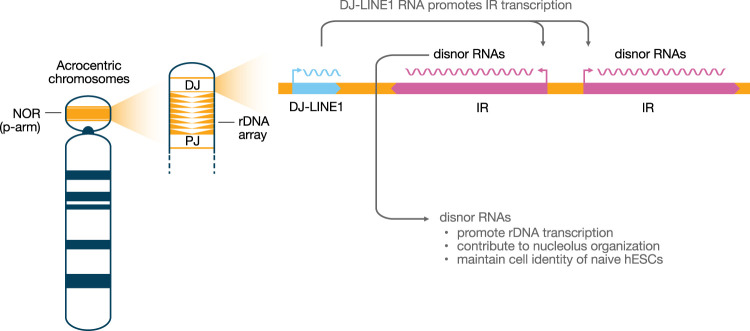
A simplified model for LINE-1 function in regulating nucleolar organization in human embryonic stem cells (hESCs). For the sake of simplicity, the nucleolar organizer region (NOR) is depicted on a metaphase chromosome and not in the context of the 3D nuclear genome. Disnor RNAs are lncRNAs encoded by the inverted repeats (IRs). Moreover, DJ-LINE1 RNA could also act independently of disnor RNAs (not shown). (rDNA) Ribosomal DNA, (DJ) distal junction, (PJ) proximal junction, (IR) inverted repeat.

Ataei et al. (2025) identified LINE-1 retrotransposons within the distal junctions of NORs (DJ-LINE1s) (Fig. 1). These LINE-1 elements are transcribed in naïve, but not in primed, human embryonic stem cells (hESCs). DJ-LINE1 DNA engages with DJ inverted repeats and, by repressing DJ-LINE1 expression, reduces chromatin accessibility near these repeats and decreases disnor transcription. These results underscore the role of DJ-LINE1s in establishing or maintaining the chromatin architecture required for disnor transcription. Accordingly, chromatin folding of DJ-LINE1 significantly changes during naïve-to-primed hESC transition, with contacts shifting from the internal part of the inverted repeats to the more distal part of the junction. Functionally, Ataei et al. (2025) also demonstrated that DJ-LINE1 expression plays a critical role in maintaining the global chromatin accessibility landscape and transcriptional regulation in naïve hESCs, preventing reversion to an 8C-like program. Previous studies have shown that direct disruption of nucleolar function in mouse ESCs induces the equivalent 2C program (Yu et al. 2021; Xie et al. 2022), suggesting that the regulatory function of DJ-LINE1 on ESC identity—and likely on early embryogenesis—may be a secondary effect of inhibiting nucleolar function. Collectively, these observations provide convincing evidence that DJ-LINE1 plays a central role in nucleolar organization and function within the NORs and thereby controls embryonic stem cell identity and transcriptional programs during human early embryogenesis.

The results from Ataei et al. (2025) have also opened several new questions and avenues for future investigations. The LINE-1 promoter is bidirectional, and its antisense activity can lead to chimeric transcripts with its adjacent sequences. It thus remains unresolved whether both sense and antisense promoter activities or only one of them contribute(s) to NOR functions. The identification of chromatin regulators and modifiers or of DJ-LINE1 RNA binders could further help to elucidate the mechanisms by which DJ-LINE1 transcripts can influence NOR activity and folding. DJ-LINE1s are present on all five human acrocentric chromosomes and likely derive from a unique retrotransposition event followed by sequence homogenization through frequent interchromosomal exchanges (van Sluis et al. 2019). Although experimentally challenging, it would be interesting to investigate whether each DJ-LINE1 element differs in its contribution to nucleolar organization and function. From an evolutionary perspective, the presence of DJ-LINE1, which belongs to the L1PA2 family and whose insertion was traced back to the split between homininae (humans, chimpanzees, and gorillas) and other great apes, raises intriguing questions about NOR regulation in nonhuman primates and other mammals. Progress in achieving telomere-to-telomere genome assemblies now provides the opportunity to sequence NORs in other mammalian species and assess whether other sequences—possibly also originating from retrotransposons—may have independently evolved to regulate nucleolus function in nonhuman species, acting similarly to DJ-LINE1. Furthermore, it is also noteworthy that knocking down DJ-LINE1 in hESCs phenocopies many characteristics observed upon global reduction of LINE-1 RNA by antisense oligonucleotides in mouse or human ESCs, including alterations in nucleolus function and cell identity (Percharde et al. 2018; Zhang et al. 2024). It remains currently unclear whether DJ-LINE1 RNA is responsible for all aspects of LINE-1 RNA activity as a lncRNA. In mice, a key role of LINE-1 RNA is to repress DUX expression, a master regulator of the 2C state. Whether DJ-LINE1 RNA similarly represses the DUX4 gene (the human functional homolog of DUX) and, reciprocally, whether DJ-LINE1-like sequences exist in mice remain to be tested.

In conclusion, the discovery by Ataei et al. (2025) provides a novel perspective on how LINE-1 retrotransposons function as genome and nuclear organizers and reinforces the notion that LINE-1 elements, although repetitive in sequence, can exhibit unique regulation and play unique roles (Lanciano et al. 2024).
